# The optimal emergency decision-making method with incomplete probabilistic information

**DOI:** 10.1038/s41598-021-02917-5

**Published:** 2021-12-03

**Authors:** Ming Fu, Lifang Wang, Bingyun Zheng, Haiyan Shao

**Affiliations:** 1 School of Management Science and Engineering, Anhui University of Finance & Economics, BengBu, 233030 Anhui China; 2 School of International Trade and Economics, Anhui University of Finance & Economics, BengBu, 233030 Anhui China; 3 School of Economics, Anhui University, HeFei, 230039 Anhui China

**Keywords:** Applied mathematics, Information technology

## Abstract

Emergencies often occur irregularly, such as infectious diseases, earthquakes, wars, floods, the diffusion and leakage of chemically toxic and harmful substances, etc. These emergencies can bring huge disasters to people, even worse, the time left for people to make critical decisions is usually very limited. When an emergency occurs, the most important thing for people is to make reasonable decisions as soon as possible to deal with the current problems, otherwise, the situation may deteriorate further. The paper proposes an emergency decision-making algorithm under the constraints of the limited time and incomplete information, the research is mainly carried out from the following aspects, firstly, we use the data structure of the hesitant fuzzy probabilistic linguistic set to collect the basic data after careful comparison, which has three advantages, (1) considering the hesitation in the decision-making process, each evaluation information is allowed to contain multiple values instead of just one value; (2) each evaluation value is followed by a probability value, which further describes the details of the evaluation information; (3) the data structure allows some probability information to be unknown, which effectively expands the application scope of the algorithm. Secondly, the maximization gap model is proposed to calculate unknown parameters, the model can distinguish alternatives with small differences. Thirdly, all the evaluation information will be aggregated by the dynamic hesitant probability fuzzy weighted arithmetic operator. Subsequently, an instance is given to illustrate the effectiveness and the accuracy of the algorithm proposed in the paper. Finally, the advantages of the proposed algorithm are further demonstrated by comparing it with other outstanding algorithms. The main contribution of the paper is that we propose the maximization gap model to obtain the unknown parameters, which can effectively and accurately distinguish alternatives with small differences.

## Introduction

In recent years, society developed rapidly, however, we also found that sometimes people’s life and social environment are becoming more and more fragile, and the frequency of unconventional emergencies is increasing^1^. Emergency management has been paid more and more attention by governments all over the world. Public emergencies mainly have some common characteristics, such as suddenness, casualties, major property losses, environmental damage and serious social adverse effects, etc. For instance, the “9.11” terrorist attacks in the United States in 2001, the SARS epidemic in China in 2003, the “5.12” earthquake in China in 2008, the Fukushima nuclear leak of Japan in 2011, the global challenge from the covid-19^2^. People gradually realize that it is necessary to formulate corresponding response plans in advance for all kinds of possible emergencies, and scientific methods for evaluating these plans are also needed, while, due to the urgency of time and the complexity of emergencies, the information obtained by people is usually incomplete and uncertain^3^, information is the basis of decision-making, therefore, how to accurately describe uncertain information and repair missing data is a tricky challenge in front of us.

Although emergencies are inevitable, with the progress of science and technology, especially the development of information technology, it is possible to minimize the losses caused by disasters^4^. One of the typical achievements is that the professor Zadeh from the University of California proposed the concept of fuzzy set in his paper “fuzzy set” in 1965^5^, different from any previous algorithms which rely on exact information, the emergence of fuzzy sets makes it possible to use mathematical methods to deal with fuzzy problems. The concept of membership degree is introduced, which can help people describe the degree that they believe an element belongs to a certain set. The theory of fuzzy sets provides a good method to deal with such complex uncertain problems. Since then, fuzzy sets have been extended to various forms for further describing uncertain information, such as intuitionistic fuzzy sets, hesitant fuzzy sets, and hesitant fuzzy linguistic sets, etc.

In 1986, Atanassov put forward the concept of the intuitionistic fuzzy set^6^ and added the non-membership degree and the hesitation degree to the membership degree in a fuzzy set. The intuitionistic fuzzy set theory provides important theoretical support for describing and dealing with the development of emergencies, however, the application scope of the intuitionistic fuzzy set still has some limitations, because it must meet the restriction that the sum of membership degree and non-membership degree should be less than or equal to 1, this restriction may cause some decision information cannot be effectively represented by the intuitionistic fuzzy set. In 2010, Torra extended the concept of the fuzzy set and proposed the concept of the hesitant fuzzy set from another perspective^7^. Considering that the decision-makers may be hesitant among several evaluation values, it is allowed to include multiple possible evaluation values in a single hesitant fuzzy set, which makes the original information can be preserved to the maximum extent. Rodriguez et al. noticed that the hesitant fuzzy set can only be used to deal with quantitative information, and he noticed that the idea of the hesitant fuzzy set could be introduced into the linguistic environment, subsequently, the concept of the hesitant fuzzy linguistic set was proposed in 2011^8^. Although the hesitant fuzzy linguistic set can exactly describe the preference information of decision-makers, while, the probability of each value in the hesitant fuzzy linguistic set is always equal to each other, which is inconsistent with the actual situation. The concept of the probabilistic hesitant fuzzy set was proposed by Pang et al. based on this consideration in 2016^9^, which allows each evaluation value has its unique probability value in the probability fuzzy set, therefore, all the original evaluation information of experts can be collected without loss. Gou and Xu^10^ further proposed basic operational laws of the probabilistic linguistic term set. In addition, Zhang et al. studied the probabilistic linguistic preference relation in 2017, considered the additive consistency of the probabilistic linguistic preference relation from the digraph perspective, and studied the consensus model by using the probabilistic linguistic preference relation^11^. Wu et al. discussed some distance, similarity, and correlation measurement methods of the probabilistic linguistic set in 2018^12^. All these research achievements have contributed to the establishment of the complete mathematical system in this field.

Recently, more and more scholars have made further discussions in this field, Yager proposed the concept of the q-order orthogonal pair fuzzy set in 2018^13^. The q-order orthogonal pair has wider constraint space and stronger modeling ability, it can describe the evaluation information of decision-makers much more exactly by expanding the space range of membership and non-membership, however, this model cannot preserve probability information. Jin et al. noticed that the existing algorithm still has some deficiencies in dealing with uncertain information, they put forward the concept of the uncertain probabilistic linguistic term set in 2019^14^, while, the algorithm cannot be closely combined with the uncertain linguistic evaluation values. Bashir et al.^15^ tried to use robust technology to solve the group decision-making problems under the uncertain linguistic environment in 2020, and novel models were designed according to the linguistic function of the linguistic terms, unfortunately, the application scope of the algorithm is too small and may not be suitable for the emergency decision-making. Ali et al. proposed an algorithm to find the most appropriate weight for the probabilistic linguistic information in 2021, regretfully, the algorithm has some limitations in the operational laws, which needs to be further studied^16^.

Although more and more research achievements have been made in recent years^17^, however, the research on the optimal emergency decision-making method with incomplete probabilistic information is only at the starting stage, especially, the research on the repair algorithm of missing data is still on the exploring period, the paper attempts to do some research in this field.

The paper discusses emergency decision-making problem from the perspective of management, the probabilistic linguistic term sets in the fuzzy mathematics is introduced to preserve original data, the maximization gap model is proposed to calculated unknown parameters, the aggregated operator in the fuzzy mathematics is used to aggregate all the evaluation information of experts, the score function converts the final evaluation information into real numbers, which is convenient for the comparison of different alternatives.

## The basic theory

This section mainly introduces the mathematical definition and properties of the hesitant fuzzy probabilistic linguistic set, as well as aggregation algorithms and comparison methods, so as to provide basic theoretical support for the optimal decision-making in the following sections.

### The probabilistic linguistic term set

The probabilistic linguistic term set (PLTS) can be defined mathematically as follows:

1 $$L_{rs} = \left\{ {\alpha_{l} (p_{l} )|\alpha_{l} \in S,p_{l} \ge 0,l = 1,2, \ldots ,m,\sum\limits_{l = 1}^{m} {p_{l} = 1} } \right\},$$

where the symbol $\alpha_{l}$ represents a series of evaluation values, the symbol $p_{l}$ represents the corresponding probability values of the evaluation values^18^, the symbol $\alpha_{l} \in S$ indicates that all the values of the $\alpha_{l}$ come from the additive linguistic term set $S$, which can be mathematically denoted as $S = \{ \alpha |\alpha = 0,1, \ldots ,2\tau \}$ and it contains all the possible values, each value $\alpha_{l}$ in the set represents an evaluation value given by an expert and the larger the value of the $\alpha$, the better the evaluation received^19^, the symbol $m$ indicates the total number of evaluation values in the probabilistic linguistic term set, the equation $\sum\nolimits_{l = 1}^{m} {p_{l} = 1}$ indicates that the sum of all probabilities is equal to 1. Each pair of the symbol $\alpha_{l} (p_{l} )$ can be called the probabilistic linguistic element (PLE), each probabilistic linguistic term set is composed of several probabilistic linguistic elements and the probabilistic linguistic element is the basic element of the probabilistic linguistic term set^20^. Let’s give a simple example to further illustrate the above content, for instance, there are two alternatives to deal with the current emergency, we must choose the most appropriate one from them as soon as possible. Firstly, we must establish the additive linguistic term set $S$, which can be described as $S = \{ 0,\;1,\;2,\;3,\;4\}$, specifically, the symbol ‘0’ indicates “terrible”, the symbol ‘1’ indicates “bad”, the symbol ‘2’ indicates “moderate”, the symbol ‘3’ indicates “good”, the symbol ‘4’ indicates “excellent”. One of probabilistic linguistic term sets can be denoted as $L_{{{\text{ij}}}} = \{ 1(0.2),\;2(0.3),\;3(0.5)\}$, which means that ‘1’, ‘2’, ‘3’ are specific evaluation values and 0.2,0.3,0.5 are their corresponding probability values given by the expert $E_{j}$ for the alternative $A_{i}$.

If all the probabilities in the probabilistic linguistic term set are equal, which can be expressed mathematically as $p_{1} = p_{2} = \cdots = p_{m} = \frac{1}{m}$, then, the probabilistic linguistic term set will be directly degraded to the hesitant fuzzy linguistic set^21^. Essentially, the probabilistic linguistic term set is an extension of the hesitant fuzzy linguistic set, and conversely, the hesitant fuzzy linguistic set is only a special case of the probabilistic linguistic term set.

The probabilistic linguistic term set can not only preserve the evaluation values but also record the occurrence probabilities, it is more suitable to describe the hesitation and uncertainty of complex systems compared with the hesitant fuzzy linguistic set^22^. It is one of the ideal mathematical support tools for emergency decision-making.

### The basic calculation methods of the probabilistic linguistic term set

It is very important to compare different PLTSs by using a unified method, Gou et al. proposed the definition of the score function to compare PLTSs^10^, the score function of the probabilistic linguistic term set can be mathematically denoted as follows:

2 $$S(L_{rs} ) = \sum\limits_{l = 1}^{m} {\alpha_{l} \cdot p_{l} } ,$$

where the symbol $\alpha_{l}$ denotes the evaluation values and the symbol $p_{l}$ denotes the corresponding probability values. Such as, the score will be $S(L_{ij} ) = 1 \times 0.2 + 2 \times 0.3 + 3 \times 0.5 = 2.3$ for the example mentioned above. Suppose there are $L_{rs}^{1}$ and $L_{rs}^{2}$, if the inequality $S(L_{rs}^{1} ) > S(L_{rs}^{2} )$ holds, then $L_{rs}^{1} > L_{rs}^{2}$, similarly, if the inequality $S(L_{rs}^{1} ) < S(L_{rs}^{2} )$ holds, then $L_{rs}^{1} < L_{rs}^{2}$. However, if the equation $S(L_{rs}^{1} ) = S(L_{rs}^{2} )$ holds, we can’t directly point out the relationship between them and we need to further calculate and compare their variances, the definition of the variance is shown as follows:

3 $$\sigma (L_{rs} ) = \left( {\sum\limits_{l = 1}^{m} {(p_{l} (\alpha_{l} - \overline{\alpha })} )^{2} } \right)^{\frac{1}{2}} \;.$$

The variance of the above example should be $\sigma (L_{ij} ) = 1.212$ according to the Eq. (3) and the specific calculation step is shown as follows:

$$\sigma (L_{ij} ) = \sqrt {0.2 \times (1 - 2.2)^{2} } + \sqrt {0.3 \times (2 - 2.2)^{2} } + \sqrt {0.5 \times (3 - 2.2)^{2} } = 1.212.$$

For the probabilistic linguistic term sets $L_{rs}^{1}$ and $L_{rs}^{2}$ when they satisfy the equation $S(L_{rs}^{1} ) = S(L_{rs}^{2} )$, if the inequality $\sigma (L_{rs}^{1} ) > \sigma (L_{rs}^{2} )$ holds, then $L_{rs}^{1} < L_{rs}^{2}$; if the inequality $\sigma (L_{rs}^{1} ) < \sigma (L_{rs}^{2} )$ holds, then $L_{rs}^{1} > L_{rs}^{2}$; if the inequality $\sigma (L_{rs}^{1} ) = \sigma (L_{rs}^{2} )$ holds, then $L_{rs}^{1} = L_{rs}^{2}$.

Let's give a simple example to illustrate the above theory. Suppose there are two PLTSs which are $L_{rs}^{1} = \{ 2(0.2),3(0.4),4(0.4)\}$ and $L_{rs}^{1} = \{ 2(0.2),3(0.4),4(0.4)\}$ respectively. The scores can be calculated according to the score function mentioned above, which are $S(L_{p}^{1} ) = S(L_{p}^{2} ) = 3.2$, We can’t directly point out which PLTS is better than the other because the scores of the two PLTSs are equal, we must further calculate their variances respectively according to the Eq. (3), which are $\sigma (L_{rs}^{1} ) = 1.169112$ and $\sigma (L_{rs}^{2} ) = 0.536656$, and the inequality $\sigma (L_{rs}^{1} ) > \sigma (L_{rs}^{2} )$ holds after comparison, therefore, we can draw the conclusion which is $L_{rs}^{1} < L_{rs}^{2}$ according to the above theory.

It is also necessary to introduce several algebraic calculation methods for PLTSs which are listed as follows:

4 $$\begin{array}{*{20}l} {(L_{rs} )^{\lambda } = \mathop \cup \limits_{{\alpha_{i} \in L_{rs} }} \{ \alpha_{i}^{\lambda } |p_{i} \} } \hfill \\ {\lambda L_{rs} = \mathop \cup \limits_{{\alpha_{i} \in L_{rs} }} \{ 1 - (1 - \alpha_{i} )^{\lambda } |p_{i} \} } \hfill \\ {L_{rs}^{1} \oplus L_{rs}^{2} = \mathop \cup \limits_{{\alpha_{i} \in L_{rs}^{1} ,\alpha_{j} \in L_{rs}^{2} }} \{ \alpha_{i} + \alpha_{j} - \alpha_{i} \alpha_{j} |p_{i} p_{j} \} } \hfill \\ {L_{rs}^{1} \otimes L_{rs}^{2} = \mathop \cup \limits_{{\alpha_{i} \in L_{rs}^{1} ,\alpha_{j} \in L_{rs}^{2} }} \{ \alpha_{i} \alpha_{j} |p_{i} p_{j} \} } \hfill \\ \end{array} .$$

In the above part of this section, we have mainly introduced the probabilistic linguistic term set with a finite number of possible elements, however, we find that it should be closer to reality when all values in a range are possible evaluation values. The definition of this kind of probabilistic linguistic term set can be denoted mathematically as the Eq. (5).

5 $$L^{\prime}_{rs} = \left\{ {\gamma_{l} |p_{l} \;\;\gamma_{l} \in [0,1],\;p_{l} \ge 0,\;l = 1,\;2, \ldots ,m,\sum\limits_{l = 1}^{m} {p_{l} = 1} } \right\}.$$

Obviously, the new definition is more concise than the older one and the algebraic calculation methods mentioned in the Eq. (4) are also applicable to the new definition^23^, we just need to replace $\gamma_{l}$ with $\alpha_{l}$. The calculation method of the score function also needs simple modification and it can be mathematically denoted as follows:

6 $$S(L^{\prime}_{rs} ) = \sum\limits_{l = 1}^{m} {\gamma_{l} \cdot p_{l} } .$$

Let’s also give a simple example to illustrate the new definition and specific calculation methods. Suppose $\lambda = 0.3$, $L_{rs}^{1{\prime}} = \{ 0.2|0.4,\;0.3|0.6\}$, $L_{rs}^{2{\prime}} = \{ 0.4|0.2,\;0.5|0.5,\;0.6|0.3\}$. The $L_{rs}^{1{\prime}}$ and $L_{rs}^{2{\prime}}$ are represented in the form of new definition, the real numbers 0.2 and 0.3 indicate evaluation values, and the real number 0.4 and 0.6 are their corresponding probability values in the PLTS $L_{rs}^{1{\prime}}$. It also has the similar structure in the PLTS $L_{rs}^{2{\prime}}$. Several simple examples are listed to illustrate the algebraic calculation method mentioned in the Eq. (4).

$$(L_{rs}^{1{\prime}} )^{\lambda } = \mathop \cup \limits_{{\gamma_{i} \in L_{rs}^{1{\prime}} }} \{ \gamma_{i}^{\lambda } |p_{i} \} = \{ 0.2^{0.2} |0.4,\;0.3^{0.2} |0.6\} = \{ 0.725|0.4,\;0.786|0.6\} ,$$

$$\lambda L_{rs}^{1{\prime}} = \mathop \cup \limits_{{\gamma_{i} \in L_{rs}^{1{\prime}} }} \{ 1 - (1 - \gamma_{i} )^{\lambda } |p_{i} \} = \{ 1 - (1 - 0.2)^{0.2} |0.4,\;1 - (1 - 0.3)^{0.2} |0.6\} = \{ 0.044|0.4,\;0.069|0.6\} ,$$

$$L_{rs}^{1{\prime}} \oplus L_{rs}^{2{\prime}} = \mathop \cup \limits_{{\gamma_{i} \in L_{rs}^{1{\prime}} ,\gamma_{j} \in L_{rs}^{2{\prime}} }} \{ \gamma_{i} + \gamma_{j} - \gamma_{i} \gamma_{j} |p_{i} p_{j} \} \; = \{ 0.52|0.08,\;0.58|0.12,\;0.6|0.2,\;0.65|0.3,\;0.68|0.12,\;0.72|0.18\} ,$$

$$L_{rs}^{1{\prime}} \otimes L_{rs}^{2{\prime}} = \mathop \cup \limits_{{\gamma_{i} \in L_{p}^{1{\prime}} ,\gamma_{j} \in L_{p}^{2{\prime}} }} \{ \gamma_{i} \gamma_{j} |p_{i} p_{j} \} = \{ 0.08|0.08,\,0.1|0.2,\,0.12|0.12,\,0.12|0.12,\,0.15|0.3,\,0.18|0.18\} .$$

Similarly, the score values of the new definition can be calculated according to the Eq. (6), the specific computation steps are shown as follows:

$$S(L_{rs}^{1{\prime}} ) = 0.2 \times 0.4 + 0.3 \times 0.6 = 0.26,$$

$$S(L_{rs}^{2{\prime}} ) = 0.4 \times 0.2 + 0.5 \times 0.5 + 0.6 \times 0.3 = 0.51.$$

### The information aggregation methods

The scattered information must be aggregated, which is also one of the important steps^24^. We will mainly introduce two common methods, which are the dynamic hesitant probability fuzzy weighted arithmetic (DHPFWA) operator and the dynamic hesitant probability fuzzy weighted geometric (DHPFWG) operator, respectively.

Suppose there are $K$ PLTSs in total which can be recorded as $L_{rs}^{1{\prime}} ,L_{rs}^{2{\prime}} , \ldots ,L_{rs}^{K^{\prime}}$, and the weight information should be given through the weight vector which can be recorded as $\omega = (\omega_{1} ,\omega_{2} , \ldots \omega_{K} )$, the weight can directly reflect the authority of the corresponding information. The weight vector satisfies the following conditions which are $\omega_{k} \in (0,1)$ and $\sum\nolimits_{k = 1}^{k} {\omega_{k} = 1}$. The DHPFWA operator can be denoted mathematically as follows:

7 $$DHPFWA(L_{rs}^{1{\prime}} ,L_{rs}^{2{\prime}} , \ldots ,L_{rs}^{K^{\prime}} ) = \mathop \oplus \limits_{k = 1}^{K} (\omega_{k} L_{rs}^{k^{\prime}} )\; = \bigcup\limits_{{\gamma_{1} \in L_{rs}^{1{\prime}} ,\gamma_{2} \in L_{rs}^{2{\prime}} , \ldots ,\gamma_{k} \in L_{rs}^{K^{\prime}} }} {\left\{ {1 - \prod\limits_{k = 1}^{K} {(1 - \gamma_{k} )^{{\omega_{k} }} } |p_{1} p_{2} \ldots p_{k} } \right\}} .$$

Let’s give a simple example to illustrate the specific calculation process of the operator. It is assumed that a total of two experts give their evaluation values for the current emergency respectively, and the evaluation information has been recorded in the PLTSs, which are denoted as $L_{rs}^{1{\prime}} = \{ 0.2|0.4,0.3|0.6\}$ and $L_{rs}^{2{\prime}} = \{ 0.4|0.2,0.5|0.5,0.6|0.3\}$. The second expert is more experienced than the first one according to the previous data analysis, therefore, the weight vector $\omega = (\omega_{1} ,\omega_{2} )$ can be set as $\omega = (0.4,0.6)$. All the evaluation information can be aggregated by the DHPFWA operator to obtain the final evaluation value, which is shown as follows:

$$\begin{aligned} DHPFWA(L_{rs}^{1{\prime}} ,L_{rs}^{2{\prime}} ) = & \mathop \oplus \limits_{k = 1}^{2} (\omega_{k} L_{rs}^{k^{\prime}} )\; = \bigcup\limits_{{\gamma_{1} \in L_{rs}^{1{\prime}} ,\gamma_{2} \in L_{rs}^{2{\prime}} }} {\left\{ {1 - \prod\limits_{k = 1}^{2} {(1 - \gamma_{k} )^{{\omega_{k} }} } |p_{1} p_{2} } \right\}} \\ \;\;\;\;\;\; = & \{ 0.327|0.08,\;0.397|0.2,\;0.472|0.12,\;0.362|0.12,\;0.428|0.3,\;0.5|0.18\} .\; \\ \end{aligned}$$

In order to better understand the calculation method of the operator, we give the detailed calculation process of the first value which is $1 - (1 - 0.2)^{0.4} \times (1 - 0.4)^{0.6} = 0.326827$, and the corresponding probability value is $0.4 \times 0.2 = 0.08$. Similarly, other values can also be calculated quickly according to the above equation. We can find that the aggregated information is also in the form of PLTS, and this is not conducive to the comparisons of multiple alternatives, therefore, we must further calculate the score value of the aggregated information, the value is 0.4239 according to the calculation method of the score value mentioned in the Eq. (6).

It is similar to the definition of the DHPFWA operator, the DHPFWG operator can be denoted mathematically as follows:

8 $$DHPFWG(L_{rs}^{1{\prime}} ,L_{rs}^{2{\prime}} , \ldots ,L_{rs}^{K^{\prime}} ) = \mathop \otimes \limits_{k = 1}^{K} (\omega_{k} L_{rs}^{k^{\prime}} )\; = \bigcup\limits_{{\gamma_{1} \in L_{rs}^{1{\prime}} ,\;\gamma_{rs} \in L_{rs}^{2{\prime}} , \ldots ,\gamma_{rs} \in L_{p}^{K^{\prime}} }} {\left\{ {\prod\limits_{k = 1}^{K} {\gamma_{k}^{{\omega_{k} }} } |p_{1} p_{2} \ldots p_{k} } \right\}} .$$

The same information aggregation problem can be also solved by the DHPFWG operator, the result is shown as follows:

$$\begin{aligned} DHPFWG(L_{rs}^{1{\prime}} ,L_{rs}^{2{\prime}} ) = & \mathop \otimes \limits_{k = 1}^{2} (\omega_{k} L_{rs}^{k^{\prime}} )\; = \bigcup\limits_{{\gamma_{1} \in L_{rs}^{1{\prime}} ,\;\gamma_{2} \in L_{rs}^{2{\prime}} ,}} {\left\{ {\prod\limits_{k = 1}^{2} {\gamma_{k}^{{\omega_{k} }} } |p_{1} p_{2} } \right\}} \;\;\; \\ \;\;\;\; = & \{ 0.303|0.08,\;0.347|0.2,\;0.387|0.12,\;0.357|0.12,\;0.408|0.3,\;0.455|0.18\} . \\ \end{aligned}$$

Similarly, the detailed calculation process of the first value will be given which is $0.2^{0.4} \times 0.4^{0.6} = {0}{\text{.303143313}}$, and the corresponding probability value is also $0.4 \times 0.2 = 0.08$. Other values can be also obtained quickly according to the above equation. We can find that the aggregated information is also in the form of PLTS, therefore, we must further calculate the score value of the aggregated information, the value is 0.3869 according to the calculation method of the score value mentioned above.

The $DHPFWA$ operator and the $DHPFWG$ operator are two common methods for the probabilistic fuzzy information aggregation, they have many similarities, while, there are also differences between them, and one of the differences is that the distinguishing ability of the $DHPFWA$ operator is greater than the $DHPFWG$ operator. The main reason is shown as follows.

The inequality (9) holds for any $L^{\prime}_{rs}$ according to the properties of functions, therefore, the inequality (10) will hold, so we can prove that the inequality $DHPFWG \le DHPFWA$ holds. The score value is 0.4239 calculated by the $DHPFWA$ operator and the score value is 0.3869 calculated by the $DHPFWG$ operator in the above instance, obviously, the inequality $0.3869 \le 0.4239$ holds.

9 $$\prod\limits_{k = 1}^{K} {\gamma_{k}^{{\omega_{k} }} } \le \sum\limits_{k = 1}^{K} {\omega_{k} } \gamma_{k} = 1 - \sum\limits_{k = 1}^{K} {\omega_{k} (1 - } \gamma_{k} ) \le 1 - \prod\limits_{k = 1}^{K} {(1 - \gamma_{k} )^{{\omega_{k} }} } ,$$

10 $$\bigcup\limits_{{\gamma_{1} \in L_{rs}^{1{\prime}} ,\gamma_{2} \in L_{rs}^{2{\prime}} , \ldots ,\gamma_{K} \in L_{rs}^{K^{\prime}} }} {\left\{ {\prod\limits_{k = 1}^{K} {\gamma_{k}^{{\omega_{k} }} } } \right\}} \le \bigcup\limits_{{\gamma_{1} \in L_{rs}^{1{\prime}} ,\gamma_{2} \in L_{rs}^{2{\prime}} , \ldots ,\gamma_{K} \in L_{rs}^{K^{\prime}} }} {\left\{ {1 - \prod\limits_{k = 1}^{K} {(1 - \gamma_{k} )^{{\omega_{k} }} } } \right\}} .$$

## The emergency decision-making algorithm with incomplete probabilistic information

The decision-making is a process in which people come up with ideas and make decisions about various events. It is a complex process including information collection, information processing, and finally making judgments and conclusions^25^. The decision-making of important issues is a scientific problem, which can be roughly divided into the following steps. Firstly, a series of alternatives will be proposed through past experiences and analyses. Secondly, some alternatives will be deleted directly through discussions and simple alternative comparisons. Thirdly, several scientific methods and algorithms must be used to deal with the remaining alternatives to find the most suitable alternative. Finally, implement the most suitable alternative and monitor the execution effect timely^26^. The third step is particularly critical and has attracted extensive attention of scholars all over the world^27^, the third step includes several sub-steps : (1) the selection of key indicators; (2) find the suitable data structure for key indicators; (3) data acquisition; (4) data normalization; (5) the selection of the most suitable aggregation algorithm; (6) information aggregation; (7) the comparisons of the aggregated values and obtain the most suitable alternative.

The emergency decision-making is one of the important branches of decision-making problems. It has the following unique characteristics compared with general decision-making problems.

i. The time for making decisions is limited^28^, and the situation will deteriorate over time if appropriate measures are not taken. This is the main feature of the emergency decision-making problem.
ii. It is usually difficult for experts to use only one unique value when evaluating an alternative, and they often hesitate among multiple values, worse still, there may be some missing data in the evaluation information. All these are due to the constraint of the limited time. Fortunately, the probabilistic linguistic term set mentioned above can preserve the original evaluation information to the greatest extent;
iii. The missing data must be repaired by suitable algorithms and only the complete data can be used for making decisions, the quality of the repair algorithm can directly influence the final solution, therefore, the data repair algorithm is one of the core problems of the paper.

### The construction of the emergency decision matrix

Suppose there are $m$ experts and $n$ alternatives. Each expert should evaluate the alternatives one by one scientifically, and then construct the emergency decision matrix which can be denoted as follows:

11 $$\begin{gathered} \;\;\;\;\;\;\;\;\;\;\;\;A_{1} \;\;\;\;\;\;A_{2} \;\;\;\;\; \cdots \;\;A_{n} \hfill \\ L = \begin{array}{*{20}c} {E_{1} } \\ {E_{2} } \\ \vdots \\ {E_{m} } \\ \end{array} \left| {\begin{array}{*{20}c} {L^{\prime}_{11} } & {L^{\prime}_{12} } & \cdots & {L^{\prime}_{1n} } \\ {L^{\prime}_{21} } & {L^{\prime}_{22} } & \cdots & {L^{\prime}_{2n} } \\ \vdots & \vdots & \vdots & \vdots \\ {L^{\prime}_{m1} } & {L^{\prime}_{m2} } & \cdots & {L^{\prime}_{mn} } \\ \end{array} } \right|\;. \hfill \\ \end{gathered}$$

### The repair of the missing data

The repair of the missing data is one of core steps, the repair algorithm will be introduced step by step in this section. Firstly, the definitions of several special probability hesitation fuzzy sets will be given^29^. The probability hesitation fuzzy element $L_{ij} = \{ 1|1\}$ indicates that the expert $E_{i}$ fully agree with the alternative $A{}_{j}$ without any doubt, the first digital symbol ‘1’ in the $L_{ij} = \{ 1|1\}$ indicates full agreement and the second digital symbol ‘1’ means no doubt. If all the elements in the emergency decision matrix are equal to $\{ 1|1\}$, then the alternative will be called the positive ideal solution^30^, which is shown as follows:

12 $$\begin{gathered} \;\;\;\;\;\;\;\;\;\;\;\;\;\;A_{p} \hfill \\ L_{1} = \begin{array}{*{20}c} {E_{1} } \\ {E_{2} } \\ \vdots \\ {E_{m} } \\ \end{array} \left| {\begin{array}{*{20}c} {\{ 1|1\} } \\ {\{ 1|1\} } \\ \vdots \\ {\{ 1|1\} } \\ \end{array} } \right|\;. \hfill \\ \end{gathered}$$

Similarly, the probability hesitation fuzzy element $L_{ij} = \{ 0|1\}$ indicates that the expert $E_{i}$ completely disagree with the alternatives $A{}_{j}$ without any doubt. The digital symbol ‘0’ in the $L_{ij} = \{ 0|1\}$ indicates total disagreement and the symbol ‘1’ in the $L_{ij} = \{ 0|1\}$ means no doubt. If all the elements in the emergency decision matrix are equal to $\{ 0|1\}$, then the alternative will be called the negative ideal solution, which is shown as follows:

13 $$\begin{gathered} \;\;\;\;\;\;\;\;\;\;\;\;\;\;A_{q} \hfill \\ L_{0} = \begin{array}{*{20}c} {E_{1} } \\ {E_{2} } \\ \vdots \\ {E_{m} } \\ \end{array} \left| {\begin{array}{*{20}c} {\{ 0|1\} } \\ {\{ 0|1\} } \\ \vdots \\ {\{ 0|1\} } \\ \end{array} } \right|\;. \hfill \\ \end{gathered}$$

The score values of the positive ideal solution and the negative ideal solution can be calculated respectively according to the Eq. (6) given in the basic part of this paper, which are shown as follows:

14 $$\begin{gathered} \;\;\;\;\;\;\;\;\;\;\;\;A_{{_{{L_{1} }} }} \hfill \\ S_{{L_{1} }} = \begin{array}{*{20}c} {E_{1} } \\ {E_{2} } \\ \vdots \\ {E_{m} } \\ \end{array} \left| {\begin{array}{*{20}c} 1 \\ 1 \\ \vdots \\ 1 \\ \end{array} } \right|\;\;, \hfill \\ \end{gathered}$$

15 $$\begin{gathered} \;\;\;\;\;\;\;\;\;\;\;\;A_{{L_{0} }} \hfill \\ S_{{L_{0} }} = \begin{array}{*{20}c} {E_{1} } \\ {E_{2} } \\ \vdots \\ {E_{m} } \\ \end{array} \left| {\begin{array}{*{20}c} 0 \\ 0 \\ \vdots \\ 0 \\ \end{array} } \right|\;\;. \hfill \\ \end{gathered}$$

Suppose the alternative $A_{x}$ is one of ordinary alternatives, and the symbol $L_{x}$ records the evaluation values of the alternative $A_{x}$, and the weight values $\omega = (\omega_{1} ,\omega_{2} , \ldots ,\omega_{M} )$ of all experts are known in advance. Firstly, the score values of the alternative $A_{x}$ can be calculated according to the evaluation values of the alternative $A_{x}$, which is shown as follows:

16 $$\begin{gathered} \;\;\;\;\;\;\;\;\;\;\;\;\;A_{x} \hfill \\ S_{{L_{x} }} = \begin{array}{*{20}c} {E_{1} } \\ {E_{2} } \\ \vdots \\ {E_{m} } \\ \end{array} \left| {\begin{array}{*{20}c} {S_{{x_{1} }} } \\ {S_{{x_{2} }} } \\ \vdots \\ {S_{{x_{m} }} } \\ \end{array} } \right|\;. \hfill \\ \end{gathered}$$

The distance between any two alternatives can be defined according to the Eq. (17), which is shown as follows:

17 $$d(A_{j} ,A_{k} ) = \left| {\sum\limits_{i}^{m} {(S_{{j_{i} }} - S_{{k_{i} }} )^{{\omega_{i} }} } } \right|\;.$$

Therefore, the distance between the positive ideal solution $A_{{L_{1} }}$ and the ordinary alternative $A_{x}$ can be calculated according to the Eq. (17), which is shown as follows:

$$d(A_{{L_{1} }} ,A_{x} ) = \left| {\sum\limits_{i}^{m} {(S_{{L_{1i} }} - S_{{x_{i} }} )^{{\omega_{i} }} } } \right| = \;\left| {\sum\limits_{i}^{m} {(1 - S_{{x_{i} }} )^{{\omega_{i} }} } } \right| = \left| {\sum\limits_{i}^{m} {(1 - S_{{x_{i} }} )^{{\omega_{i} }} } } \right| = \sum\limits_{i}^{m} {(1 - S_{{x_{i} }} )^{{\omega_{i} }} } .$$

Similarly, the distance between the negative ideal solution $A_{{L_{0} }}$ and the ordinary alternative $A_{x}$ can be also calculated according to the Eq. (17), which is shown as follows:

$$d(A_{{L_{0} }} ,A_{x} ) = \left| {\sum\limits_{i}^{m} {(S_{{L_{0i} }} - S_{{x_{i} }} )^{{\omega_{i} }} } } \right| = \;\left| {\sum\limits_{i}^{m} {(0 - S_{{x_{i} }} )^{{\omega_{i} }} } } \right| = \left| {\sum\limits_{i}^{m} {(0 - S_{{x_{i} }} )^{{\omega_{i} }} } } \right| = \left| {\sum\limits_{i}^{m} {( - S_{{x_{i} }} )^{{\omega_{i} }} } } \right| = \sum\limits_{i}^{m} {(S_{{x_{i} }}^{{\omega_{i} }} )} .$$

The traditional TOPSIS(Technique for Order Preference by Similarity to an Ideal Solution) algorithm is an outstanding algorithm, which sorts alternatives according to the distances between alternatives and ideal solutions^31^, if the alternative is closest to the optimal solution and also furthest away from the worst solution, then the alternative will be the most suitable solution, while, the traditional TOPSIS algorithm cannot directly deal with the data which are in the form of the probabilistic linguistic term sets. Inspired by the traditional TOPSIS method^32^, the expected level of the alternative $A_{x}$ is defined as shown in the Eq. (18).

18 $$\begin{gathered} \sigma (A_{x} ) = \frac{{(1 - \theta ) \cdot d(A_{{L_{0} }} ,A_{x} )}}{{\theta \cdot d(A_{{L_{1} }} ,A_{x} ) + (1 - \theta ) \cdot d(A_{{L_{0} }} ,A_{x} )}}\;\; \hfill \\ \;\;\;\;\;\;\;\;\; = \frac{{(1 - \theta ) \cdot \sum\limits_{i}^{m} {(S_{{x_{i} }}^{{\omega_{i} }} )} }}{{\theta \cdot \sum\limits_{i}^{m} {(1 - S_{{x_{i} }} )^{{\omega_{i} }} } + (1 - \theta ) \cdot \sum\limits_{i}^{m} {(S_{{x_{i} }}^{{\omega_{i} }} )} }}. \hfill \\ \end{gathered}$$

The parameter $\theta$ denotes the risk preference of decision-makers. Decision-makers are more willing to take risks if the $\theta$ is set between 0.5 and 1; while, decision-makers prefer to pursue certainty if the $\theta$ is set between 0 and 0.5.

In order to repair the missing data in the emergency decision matrix the expected model is proposed, which is shown as follows:

19 $$\begin{gathered} {\text{model 1:}} \hfill \\ \zeta = \max \left( {\sum\limits_{\alpha = 1}^{n - 1} {\sum\limits_{\beta = 1,\partial < \beta }^{n} {(|\tau_{\alpha } - \tau_{\beta } |)} } } \right)\;\; \hfill \\ \;\;s.t.\;\; \hfill \\ \;\;\sigma (A_{i} ) \ge \tau_{i} \;\;i = 1,2, \ldots n\;\;\;\;\;\;\;\;\;\;\;\;\;\;\;\; \hfill \\ \;\;\omega = {(}\omega_{1} {,}\omega_{2} {,} \ldots \omega_{m} {)} \hfill \\ \, \sum\limits_{i = 1}^{m} {\omega_{i} } = 1. \hfill \\ \end{gathered}$$

The model is mainly based on the idea of maximizing the expected gap between any two alternatives, and it is a linear model with several unknown parameters, it can be solved efficiently with the help of the lingo software^33^. The missing data can be obtained by solving the model 1. The emergency decision matrix with complete information will be obtained after this step.

### Information aggregation and alternatives ranking

The evaluation information of all experts will be aggregated in this step. Common aggregation algorithms cannot complete this work because of all the evaluation information adopts the data structure of the probability hesitation fuzzy set. Fortunately, the DHPFWA operator and the DHPFWG operator mentioned in the Eqs. (7) and (8) above can aggregate this kind of information efficiently. However, through the calculation of operators, multiple probability hesitation fuzzy elements rather than real numbers will be obtained, and they can’t compare with each other directly. Then, the score function will further process them according to the calculation Eq. (6) mentioned in the basic theory above. At this time, each alternative will get a final evaluation value, which can be directly compared with each other, alternatives will be sorted according to this values, and then the most suitable alternative will be found. An instance with detailed steps will be given in the next section.

## An instance of the emergency decision-making with incomplete information

Currently, the Covid-19 epidemic is still spreading, the work of preventing the spread of the epidemic is still facing severe challenges. Sometimes, the number of infected people in a certain area may surge in a short time, the local authorities must make appropriate decisions and take corresponding measures immediately to prevent the further spread of the disease, what's worse, due to the limited time, the information available for making decisions is incomplete, which is a typical emergency decision-making problem with incomplete information obviously.

### The evaluation of alternatives

Suppose, there are four experts denoted as $E = (E_{1} ,\;E_{2} ,\;E_{3} ,\;E_{4} )$ and three alternatives denoted as $A = (A_{1} ,\;A_{2} ,\;A_{3} )$ at present. The experts must evaluate the alternatives individually and find the most suitable alternative as soon as possible. The weight values of the four experts are set to $\omega = (0.21,\;0.24,\;0.27,\;0.28)$ respectively according to their authorities in this field. Experts may hesitate among multiple values when evaluating alternatives, therefore, all the evaluation values adopt the data structure of the hesitant fuzzy linguistic set. After all experts evaluate the alternatives, the emergency evaluation decision matrix will be constructed, as shown in the Table 1.

**Table 1:** The emergency evaluation decision matrix with incomplete information.

Alternatives experts	*A*_1_	*A*_2_	*A*_2_
$E_{1}$	{0.88|0.4, 0.82|0.6}	{0.86|0.3, 0.83|0.7}	{0.85|0.2 − x, 0.83|0.8, 0.80|x}
$E_{2}$	{0.87|0.5, 0.84|y, 0.80|0.5 − y}	{0.84|0.6, 0.83|0.4}	{0.85|0.7, 0.82|0.3}
$E_{3}$	{0.85|0.4, 0.82|0.6}	{0.88|m, 0.87|0.6 − m, 0.82|0.4}	{0.84|0.5, 0.81|0.5}
$E_{4}$	{0.86|0.5, 0.81|0.5}	{0.87|0.2, 0.81|0.8}	{0.88|n, 0.87|0.6 − n, 0.82|0.4}

We find that there are some unknown parameters in the emergency evaluation decision matrix, such as, $L^{\prime}_{21} =${0.87|0.5, 0.84|y, 0.80|0.5 − y}, it means that three evaluation values are given by the expert $E_{2}$ for the alternative $A_{1}$ and they are 0.87, 0.84 and 0.80 respectively, the probability of the first evaluation value 0.87 is 0.5, while, the probability of the second evaluation value 0.84 is uncertain, since the sum of all probability values of any PLE is 1, therefore, the probability value of the third evaluation value can be calculated, which is 0.5 − y.

### The repair of the missing data

Firstly, we can calculate the score values of the emergency evaluation values according to the Eq. (6) mentioned above, the score values are shown in the Table 2.

**Table 2:** The score values with unknown parameters.

Alternatives experts	*A*_1_	*A*_2_	*A*_3_
$E_{1}$	0.844	0.839	0.834 − 0.05x
$E_{2}$	0.835 + 0.04y	0.836	0.841
$E_{3}$	0.832	0.85 + 0.01 m	0.825
$E_{4}$	0.835	0.822	0.85 + 0.01n

Then, the model will be established according to the model given in the Eq. (18), and the specific form is shown as follows:

$$\begin{array}{*{20}l} {\text{model}}: \hfill \\ \zeta = \max ({|}\tau_{1} { - }\tau_{2} {|} + {|}\tau_{1} { - }\tau_{3} {| + |}\tau_{2} { - }\tau_{3} {|}) \hfill \\ s.t. \hfill \\ \frac{{(1 - \theta ) \times [0.844^{{\omega_{1} }} + (0.835 + 0.04 \times y)^{{\omega_{2} }} + 0.832^{{\omega_{3} }} + 0.835^{{\omega_{4} }} ]}}{{\theta \times [(0.156)^{{\omega_{1} }} + (0.165 - 0.04 \times y)^{{\omega_{2} }} + 0.168^{{\omega_{3} }} + 0.165^{{\omega_{4} }} ] + (1 - \theta ) \times [0.844^{{\omega_{1} }} + (0.835 + 0.04 \times y)^{{\omega_{2} }} + 0.832^{{\omega_{3} }} + 0.835^{{\omega_{4} }} ]}} \ge \tau_{1} \hfill \\ \frac{{(1 - \theta ) \times [0.839^{{\omega_{1} }} + 0.836^{{\omega_{2} }} + (0.85 + 0.01 \times m)^{{\omega_{3} }} + 0.822^{{\omega_{4} }} ]}}{{\theta \times [0.161^{{\omega_{1} }} + 0.164^{{\omega_{2} }} + (0.15 - 0.01 \times m)^{{\omega_{3} }} + 0.178^{{\omega_{4} }} ] + (1 - \theta ) \times [0.839^{{\omega_{1} }} + 0.836^{{\omega_{2} }} + (0.85 + 0.01 \times m)^{{\omega_{3} }} + 0.822^{{\omega_{4} }} ]}} \ge \tau_{2} \hfill \\ \frac{{(1 - \theta ) \times [(0.834 - 0.05 \times x)^{{\omega_{1} }} + 0.841^{{\omega_{2} }} + 0.825^{{\omega_{3} }} + (0.85 + 0.01 \times n)^{{\omega_{4} }} ]}}{{\theta \times [(0.166 + 0.05 \times x)^{{\omega_{1} }} + 0.159^{{\omega_{2} }} + 0.175^{{\omega_{3} }} + (0.15 - 0.01 \times n)^{{\omega_{4} }} ] + (1 - \theta ) \times [(0.834 - 0.05 \times x)^{{\omega_{1} }} + 0.841^{{\omega_{2} }} + 0.825^{{\omega_{3} }} + (0.85 + 0.01 \times n)^{{\omega_{4} }} ]}} \ge \tau_{3} \hfill \\ \omega = (0.21,0.24,0.27,0.28); \hfill \\ \theta = 0.6; \hfill \\ 0 \le m \le 0.6; \hfill \\ 0 \le n \le 0.6; \hfill \\ 0 \le x \le 0.2; \hfill \\ 0 \le y \le 0.5. \hfill \\ \end{array}$$

The model can be solved by the lingo software efficiently, and all the unknown parameters can be obtained, and they are $m = 0.6$, $n = 0.3$, $x = 0.2$, $y = 0.5$ respectively. Subsequently, the complete score values and the emergency evaluation matrix will be obtained, they are shown in the Tables 3 and 4 respectively.

**Table 3:** The complete score values of the emergency evaluation matrix.

Alternatives experts	*A*_1_	*A*_2_	*A*_3_
$E_{1}$	0.844	0.839	0.824
$E_{2}$	0.855	0.836	0.841
$E_{3}$	0.832	0.856	0.825
$E_{4}$	0.835	0.822	0.853

**Table 4:** The complete emergency evaluation decision matrix.

Alternatives experts	*A*_1_	*A*_2_	*A*_3_
$E_{1}$	{0.88|0.4, 0.82|0.6}	{0.86|0.3, 0.83|0.7}	{0.83|0.8, 0.80|0.2}
$E_{2}$	{0.87|0.5, 0.84|0.5}	{0.84|0.6, 0.83|0.4}	{0.85|0.7, 0.82|0.3}
$E_{3}$	{0.85|0.4, 0.82|0.6}	{0.88|0.6, 0.82|0.4}	{0.84|0.5, 0.81|0.5}
$E_{4}$	{0.86|0.5, 0.81|0.5}	{0.87|0.2, 0.81|0.8}	{0.88|0.3, 0.87|0.3, 0.82|0.4}

### The most suitable alternative

We found that the most suitable alternative could not be obtained by simply comparing the alternatives in the Table 3, therefore, we must aggregate the evaluation information comprehensively through appropriate algorithms. Both the DHPFWA and the DHPFWG operators mentioned above can process this problem efficiently, and we only give the specific implementation process of adopting the DHPFWA operator, the execution process by using the DHPFWG operator is similar to the DHPFWA operator. The information aggregation values of alternatives can be obtained according to the Eq. (7), which are shown as follows:

$$DHPFWA(A_{1} ) = \mathop \oplus \limits_{k = 1}^{4} (\omega_{k} L_{k1} ) = \left\{ \begin{gathered} 0.8643|0.04,0.8522|0.04,0.8575|0.06,0.8448|0.06 \hfill \\ 0.8574|0.04,0.8447|0.04,0.8502|0.06,0.8368|0.06 \hfill \\ 0.8523|0.06,0.8391|0.06,0.8448|0.09,0.8310|0.09 \hfill \\ 0.8447|0.06,0.8309|0.06,0.8369|0.09,0.8223|0.09 \hfill \\ \end{gathered} \right\},$$

$$DHPFWA(A_{2} ) = \mathop \oplus \limits_{k = 1}^{4} (\omega_{k} L_{k2} ) = \left\{ \begin{gathered} 0.8642|0.0216,0.8490|0.0864,0.8485|0.0144,0.8315|0.0576 \hfill \\ 0.8622|0.0144,0.8467|0.0576,0.8462|0.0096,0.8290|0.0384 \hfill \\ 0.8585|0.0504,0.8427|0.2016,0.8422|0.0336,0.8245|0.1344 \hfill \\ 0.8565|0.0336,0.8404|0.1344,0.8398|0.0224,0.8219|0.0896 \hfill \\ \end{gathered} \right\},$$

$$DHPFWA(A_{3} ) = \mathop \oplus \limits_{k = 1}^{4} (\omega_{k} L_{k3} ) = \left\{ \begin{gathered} 0.8528|0.084,0.8495|0.084,0.8458|0.084,0.8423|0.084 \hfill \\ 0.8462|0.036,0.8427|0.036,0.8389|0.036,0.8352|0.036 \hfill \\ 0.8477|0.021,0.8442|0.021,0.8404|0.021,0.8368|0.021 \hfill \\ 0.8409|0.009,0.8373|0.009,0.8333|0.009,0.8295|0.009 \hfill \\ 0.8351|0.112,0.8273|0.112,0.8277|0.048,0.8195|0.048 \hfill \\ 0.8294|0.028,0.8213|0.028,0.8217|0.012,0.8133|0.012 \hfill \\ \end{gathered} \right\}.$$

We found that it is still impossible to rank alternatives because the values are not real numbers, therefore, the score values will be further calculated which are shown as follows:

$$S(DHPFWA(A_{1} )) = 0.842285,$$

$$S(DHPFWA(A_{2} )) = 0.839706,$$

$$S(DHPFWA(A_{3} )) = 0.837769.$$

We can find that these values are very close to each other, which indicates that it is difficult to rank these alternatives by general algorithms from another point of view, meanwhile, it also shows that the algorithm proposed in this paper can identify subtle differences. The most suitable alternative will be the first alternative in this instance because the inequality $S(A_{1} ) > S(A_{2} ) > S(A_{3} )$ holds, the alternative $A_{1}$ must be implemented as soon as possible and the execution effect must be continuously recorded.

## The comparison of different algorithms

Different algorithms will be used to deal with the same problem proposed in the previous section of this paper, and try to find out the advantages and disadvantages of these algorithms.

### The hesitant fuzzy algorithm

The hesitant fuzzy algorithm is also one of the outstanding algorithms to deal with decision-making problems^34^. The hesitant fuzzy algorithm is similar to the probabilistic hesitant fuzzy algorithm proposed in the paper, however, the main difference is that it cannot deal with the probability information, therefore, the probability information has to be discarded when the hesitant fuzzy algorithm is adopted, and after the probability information is discarded, the emergency evaluation decision matrix without probability information is shown in the Table 5.

20 $$HFWA_{\omega } (h_{1} ,h_{2} , \ldots ,h_{m} ) = \mathop \oplus \limits_{j = 1}^{m} (\omega_{j} h_{j} ) = \cup_{{\gamma_{1} \in h_{1} ,\gamma_{2} \in h_{2} , \ldots ,\gamma_{m} \in h_{m} }} \left\{ {1 - \prod\limits_{j = 1}^{m} {(1 - \gamma_{j} )^{{\omega_{j} }} } } \right\},$$

21 $$s^{\prime}(h) = \frac{1}{{l_{h} }}\sum\limits_{\gamma \in h} \gamma .$$

**Table 5:** The emergency evaluation decision matrix without probability information.

Alternatives experts	*A*_1_	*A*_2_	*A*_3_
$E_{1}$	{0.88, 0.82}	{0.86, 0.83}	{0.85, 0.83, 0.80}
$E_{2}$	{0.87, 0.84, 0.80}	{0.84, 0.83}	{0.85, 0.82}
$E_{3}$	{0.85, 0.82}	{0.88, 0.87, 0.82}	{0.84, 0.81}
$E_{4}$	{0.86, 0.81}	{0.87, 0.81}	{0.88, 0.87, 0.82}

All the information for each alternative can be aggregated according to the Eq. (20) and we can obtain several complex hesitant fuzzy sets, which cannot be used directly for alternative ranking. The calculation results are shown respectively as follows:

$$HFWA_{1} = \mathop \oplus \limits_{j = 1}^{m} (\omega_{j} h_{j} ) = \left( {\begin{array}{*{20}c} {0.8643} & {0.8522} & {0.8575} & {0.8448} & {0.8574} & {0.8447} \\ {0.8502} & {0.8368} & {0.8523} & {0.8391} & {0.8448} & {0.8310} \\ {0.8447} & {0.8309} & {0.8369} & {0.8223} & {0.8496} & {0.8361} \\ {0.8420} & {0.8279} & {0.8362} & {0.8216} & {0.8279} & {0.8126} \\ \end{array} } \right),$$

$$HFWA_{2} = \mathop \oplus \limits_{j = 1}^{m} (\omega_{j} h_{j} ) = \left( {\begin{array}{*{20}c} {0.8642} & {0.8490} & {0.8612} & {0.8457} & {0.8622} & {0.8467} \\ {0.8592} & {0.8434} & {0.8585} & {0.8427} & {0.8554} & {0.8392} \\ {0.8565} & {0.8404} & {0.8533} & {0.8369} & {0.8485} & {0.8315} \\ {0.8462} & {0.8290} & {0.8422} & {0.8245} & {0.8398} & {0.8219} \\ \end{array} } \right),$$

$$HFWA_{3} = \mathop \oplus \limits_{j = 1}^{m} (\omega_{j} h_{j} ) = \left( {\begin{array}{*{20}c} {0.8566} & {0.8528} & {0.8477} & {0.8534} & {0.8495} & {0.8442} \\ {0.8394} & {0.8351} & {0.8294} & {0.8498} & {0.8458} & {0.8404} \\ {0.8464} & {0.8423} & {0.8368} & {0.8317} & {0.8273} & {0.8213} \\ {0.8502} & {0.8462} & {0.8409} & {0.8468} & {0.8427} & {0.8373} \\ {0.8322} & {0.8277} & {0.8217} & {0.8431} & {0.8389} & {0.8333} \\ {0.8395} & {0.8352} & {0.8295} & {0.8242} & {0.8195} & {0.8133} \\ \end{array} } \right).$$

The score values of the hesitant fuzzy sets can be further calculated according to the Eq. (21), and the result is real number, which is convenient for comparison, the final results can be obtained after the above two steps, which are shown as follows:

$$S^{\prime}(A_{1} ) = 0.840165,$$

$$S^{\prime}(A_{2} ) = 0.845749,$$

$$S^{\prime}(A_{3} ) = 0.838112.$$

It can be concluded that the inequality $S^{\prime}(A_{2} ) > S^{\prime}(A_{1} ) > S^{\prime}(A_{3} )$ holds according to the above calculation results. We find that the ranking of alternatives obtained by using this algorithm is completely different from the algorithm proposed in this paper. The second alternative $A_{2}$ is the most suitable solution by using the hesitant fuzzy algorithm, while, the first alternative $A_{1}$ is the most suitable solution by using the algorithm proposed in this paper. We believe that the algorithm proposed in this paper is more reliable, that is because that the valuable probability information objectively given by experts has been completely discarded in the hesitant fuzzy algorithm, in other words, part important information didn’t play any role, thus, the calculation result is untrusted. On the contrary, it is also further proved the probability information can plays an important role in the decision-making. So, compared with the hesitant fuzzy algorithm, the probabilistic hesitant fuzzy algorithm can more accurately describe the actual ideas of experts.

### The maximum expected level algorithm

The unknown parameters can also be calculated by the maximum expected level algorithm^35^ and the main idea of the algorithm is to maximize the comprehensive evaluation result, the model can be described mathematically as follows:

22 $$\begin{array}{*{20}l} {\text{model}}\;{2:} \hfill \\ \zeta = \max (\tau_{1} + \tau_{2} + \cdots + \tau_{N} )\;\; \hfill \\ \;\;s.t.\;\; \hfill \\ \;\;\sigma (A_{i} ) \ge \tau_{i} \;\;i = 1,2, \ldots N \hfill \\ \;\;\omega = {(}\omega_{1} {,}\omega_{2} {,} \ldots \omega_{M} {)} \hfill \\ \, \sum\limits_{i = 1}^{M} {\omega_{i} } = 1. \hfill \\ \end{array}$$

Similarly, we can construct a linear model according to the Table 1 and the model 2, the specific model is shown as follows:

$$\begin{gathered} {\text{model}}: \hfill \\ \zeta = \max (\tau_{1} { + }\tau_{2} { + }\tau_{3} ) \hfill \\ s.t. \hfill \\ \frac{{(1 - \theta ) \times [0.844^{{\omega_{1} }} + (0.835 + 0.04 \times y)^{{\omega_{2} }} + 0.832^{{\omega_{3} }} + 0.835^{{\omega_{4} }} ]}}{{\theta \times [(0.156)^{{\omega_{1} }} + (0.165 - 0.04 \times y)^{{\omega_{2} }} + 0.168^{{\omega_{3} }} + 0.165^{{\omega_{4} }} ] + (1 - \theta ) \times [0.844^{{\omega_{1} }} + (0.835 + 0.04 \times y)^{{\omega_{2} }} + 0.832^{{\omega_{3} }} + 0.835^{{\omega_{4} }} ]}} \ge \tau_{1} \hfill \\ \frac{{(1 - \theta ) \times [0.839^{{\omega_{1} }} + 0.836^{{\omega_{2} }} + (0.85 + 0.01 \times m)^{{\omega_{3} }} + 0.822^{{\omega_{4} }} ]}}{{\theta \times [0.161^{{\omega_{1} }} + 0.164^{{\omega_{2} }} + (0.15 - 0.01 \times m)^{{\omega_{3} }} + 0.178^{{\omega_{4} }} ] + (1 - \theta ) \times [0.839^{{\omega_{1} }} + 0.836^{{\omega_{2} }} + (0.85 + 0.01 \times m)^{{\omega_{3} }} + 0.822^{{\omega_{4} }} ]}} \ge \tau_{2} \hfill \\ \frac{{(1 - \theta ) \times [(0.834 - 0.05 \times x)^{{\omega_{1} }} + 0.841^{{\omega_{2} }} + 0.825^{{\omega_{3} }} + (0.85 + 0.01 \times n)^{{\omega_{4} }} ]}}{{\theta \times [(0.166 + 0.05 \times x)^{{\omega_{1} }} + 0.159^{{\omega_{2} }} + 0.175^{{\omega_{3} }} + (0.15 - 0.01 \times n)^{{\omega_{4} }} ] + (1 - \theta ) \times [(0.834 - 0.05 \times x)^{{\omega_{1} }} + 0.841^{{\omega_{2} }} + 0.825^{{\omega_{3} }} + (0.85 + 0.01 \times n)^{{\omega_{4} }} ]}} \ge \tau_{3} \hfill \\ \omega = (0.21,0.24,0.27,0.28); \hfill \\ \theta = 0.6; \hfill \\ 0 \le m \le 0.6; \hfill \\ 0 \le n \le 0.6; \hfill \\ 0 \le x \le 0.2; \hfill \\ 0 \le y \le 0.5. \hfill \\ \end{gathered}$$

The model can be solved with the help of the lingo software, therefore, the values of the unknown parameters are obtained after calculation, which are $m = 0.6$, $n = 0.6$, $x = 0$, $y = 0.5$, then, the complete evaluation decision matrix can be obtained which is shown in the Table 6.

**Table 6:** The complete evaluation decision matrix repaired by the maximum expected level algorithm.

Alternatives experts	*A*_1_	*A*_2_	*A*_3_
$E_{1}$	{0.88|0.4, 0.82|0.6}	{0.86|0.3, 0.83|0.7}	{0.85|0.2, 0.83|0.8}
$E_{2}$	{0.87|0.5, 0.84|0.5}	{0.84|0.6, 0.83|0.4}	{0.85|0.7, 0.82|0.3}
$E_{3}$	{0.85|0.4, 0.82|0.6}	{0.88|0.6, 0.82|0.4}	{0.84|0.5, 0.81|0.5}
$E_{4}$	{0.86|0.5, 0.81|0.5}	{0.87|0.2, 0.81|0.8}	{0.88|0.6, 0.82|0.4}

The information can be comprehensively aggregated according to the Eq. (7), and the aggregated values are shown as follows:

$$DHPFWA^{\prime\prime}(A_{1} ) = \mathop \oplus \limits_{k = 1}^{4} (\omega_{k} L_{k1} ) = \left\{ \begin{gathered} 0.8643|0.04,0.8522|0.04,0.8575|0.06,0.8448|0.06 \hfill \\ 0.8574|0.04,0.8447|0.04,0.8502|0.06,0.8368|0.06 \hfill \\ 0.8523|0.06,0.8391|0.06,0.8448|0.09,0.8310|0.09 \hfill \\ 0.8447|0.06,0.8309|0.06,0.8369|0.09,0.8223|0.09 \hfill \\ \end{gathered} \right\},$$

$$DHPFWA^{\prime\prime}(A_{2} ) = \mathop \oplus \limits_{k = 1}^{4} (\omega_{k} L_{k2} ) = \left\{ \begin{gathered} 0.8642|0.0216,0.8490|0.0864,0.8485|0.0144,0.8315|0.0576 \hfill \\ 0.8622|0.0144,0.8467|0.0576,0.8462|0.0096,0.8290|0.0384 \hfill \\ 0.8585|0.0504,0.8427|0.2016,0.8422|0.0336,0.8245|0.1344 \hfill \\ 0.8565|0.0336,0.8404|0.1344,0.8398|0.0224,0.8219|0.0896 \hfill \\ \end{gathered} \right\},$$

$$DHPFWA^{\prime\prime}(A_{3} ) = \mathop \oplus \limits_{k = 1}^{4} (\omega_{k} L_{k3} ) = \left\{ \begin{gathered} 0.8566|0.042,0.8394|0.028,0.8498|0.042,0.8317|0.028 \hfill \\ 0.8502|0.018,0.8322|0.012,0.8431|0.018,0.8242|0.012 \hfill \\ 0.8528|0.168,0.8351|0.112,0.8458|0.168,0.8273|0.112 \hfill \\ 0.8462|0.072,0.8277|0.048,0.8389|0.072,0.8195|0.048 \hfill \\ \end{gathered} \right\}.$$

Similarly, the score values can be further calculated according to the Eq. (6). The final calculation results are listed as follows:

$$S^{\prime\prime}(A_{1} ) = 0.842285,$$

$$S^{\prime\prime}(A_{2} ) = 0.839706,$$

$$S^{\prime\prime}(A_{3} ) = 0.840757.$$

Therefore, the ranking of alternatives is $S^{\prime\prime}(A_{1} ) > S^{\prime\prime}(A_{3} ) > S^{\prime\prime}(A_{2} )$, which is different from the result of the algorithm proposed in the paper and the hesitant fuzzy algorithm mentioned earlier.

However, the algorithm has two shortcomings through comprehensive analysis, the first shortcoming is that all the evaluation information tends to the maximum values and directly ignores the intermediate values, which will lead to the loss of objective information; another shortcoming is that, compared with the algorithm proposed in the paper, the distances between the final score values are smaller, which are not conducive to the ranking of alternatives. Specifically, the distances are 0.002579 and 0.001937 respectively when adopting the algorithm proposed in the paper, while, the distances are 0.001528 and 0.001051 respectively when adopting the maximum expected level algorithm.

We think that the most suitable alternative is $A_{1}$ after the above analyses. While, we also realize that the loss of any detailed information may result in completely different ranking results of alternatives. We believe that the algorithm proposed in the paper is more objective after algorithm comparisons.

## Conclusions

In today's society, emergencies occur from time to time, resulting in a large number of property losses and casualties. If appropriate measures are not taken to deal with these problems, the situation will often get worse and worse over time. Therefore, when a disaster occurs, it is the primary problem to make a scientific and reasonable decision as soon as possible. The emergency decision-making is the main research content of this paper and has always been a hot issue in the academic circle. This paper mainly discusses this problem from the perspective of management and tries to propose an efficient method to solve this problem.

High-quality data is the basis for making appropriate decisions, while, how to obtain high-quality data is the first problem we need to solve. In addition to carefully and objectively evaluating each alternative, it is particularly important to choose an appropriate data structure to preserve original evaluation detail information. After careful comparison, we adopt the data structure of the hesitant fuzzy probabilistic linguistic set, which mainly has three unique advantages, firstly, it can save multiple possible values in one evaluation value, and fully consider the hesitation of experts in the evaluation process; secondly, each possible evaluation value is followed by a corresponding probability value; thirdly, in order to further simulate the actual situation, some probability values are allowed to be unknown. Compared with all other data structures known by the authors, the hesitant fuzzy probabilistic linguistic set can indeed preserve the original evaluation data of experts to the greatest extent.

The repair method of the missing data is also one of the core problems of this paper. The quality of the repair algorithm is very critical, the experiments in the previous section show that even small differences in decision information may directly affect the ranking of alternatives. The paper proposes a maximization gap algorithm, it can maximize the distances between alternatives under all constraints, all unknown parameters can be obtained quickly through this repair algorithm. Subsequently, the information aggregation algorithm will aggregate all the evaluation information, the method used in the paper can not only consider the evaluation values, but also consider the authorities of experts, and then the score values will be calculated, all the alternatives can be ranked according to this values.

We compare the algorithm with the hesitant fuzzy algorithm and the maximum expected level algorithm respectively. The probability information is not considered totally in the hesitant fuzzy algorithm, in other words, each probability is equal to each other in this algorithm, and it can be regarded as a simple form of the algorithm proposed in this paper. The maximum expected level algorithm can maximize the score value of each alternative, however, the evaluation value tends to the maximum value which makes the intermediate values easy to be ignored, and that means some objective evaluation information given by experts may be directly ignored. Detailed implementation steps are given through an instance and algorithm comparisons are also given in the previous section, we find that even small differences in probability values may lead to completely different rankings of alternatives, and the algorithm proposed in this paper can indeed obtain the optimal solution efficiently and accurately.

At the same time, we also realize that emergencies often develop dynamically. The current decision-making algorithm mainly makes optimal decisions based on the current data, decisions need to be made continuously, while, and the dynamic decision-making can be automatically adjusted according to the development of emergency. The method proposed in this paper does not support dynamic optimal decision-making, which will be the focus of our team's next research.
